# Genetic analysis of albinism caused by compound heterozygous mutations of the *OCA2* gene in a Chinese family

**DOI:** 10.1186/s41065-024-00312-4

**Published:** 2024-02-06

**Authors:** Yanan Wang, Yujie Chang, Mingya Gao, Weiwei Zang, Xiaofei Liu

**Affiliations:** Genetics and Prenatal Diagnosis Department, Luoyang Maternal and Child Health Hospital, Luoyang, China

**Keywords:** Albinism, *OCA2* gene, De novo mutation, Compound heterozygous mutations

## Abstract

**Background:**

Oculocutaneous albinism (OCA) is a group of rare genetic disorders characterized by a reduced or complete lack of melanin in the skin, hair, and eyes. Patients present with colorless retina, pale pink iris, and pupil, and fear of light. The skin, eyebrows, hair, and other body hair are white or yellowish-white. These conditions are caused by mutations in specific genes necessary for the production of melanin. OCA is divided into eight clinical types (OCA1-8), each with different clinical phenotypes and potential genetic factors. This study aimed to identify the genetic causes of non-syndromic OCA in a Chinese Han family.

**Methods:**

We performed a comprehensive clinical examination of family members, screened for mutation loci using whole exome sequencing (WES) technology, and predicted mutations using In silico tools.

**Results:**

The patient’s clinical manifestations were white skin, yellow hair, a few freckles on the cheeks and bridge of the nose, decreased vision, blue iris, poorly defined optic disk borders, pigmentation of the fundus being insufficient, and significant vascular exposure. The WES test results indicate that the patient has compound heterozygous mutations in the *OCA2* gene (c.1258G > A (p.G420R), c.1441G > A (p.A481T), and c.2267-2 A > C), respectively, originating from her parents. Among them, c.1258G > A (p.G420R) is a de novo mutation with pathogenic. Our analysis suggests that compound heterozygous mutations in the *OCA2* gene are the primary cause of the disease in this patient.

**Conclusions:**

The widespread application of next-generation sequencing technologies such as WES in clinical practice can effectively replace conventional detection methods and assist in the diagnosis of clinical diseases more quickly and accurately. The newly discovered c.1258G > A (p.G420R) mutation can update and expand the gene mutation spectrum of OCA2-type albinism.

**Supplementary Information:**

The online version contains supplementary material available at 10.1186/s41065-024-00312-4.

Albinism is a hereditary disease caused by deficiency or impaired synthesis of melanin in the skin and accessory organs due to tyrosinase deficiency or hypofunction. The patient has an unpigmented retina, photophobia, pale pink or pale blue iris and pupil, and white or yellowish-white skin, eyebrows, and hair [1].

The prevalence of OCA varies greatly by race. The worldwide prevalence of OCA is about 1/17,000, and the mutation carrier rate is about 1/65. The prevalence of OCA in the Chinese Han population is about 1/18,000, and the mutation carrier rate is about 1/70 [2, 3]. The higher prevalence puts a great burden on the patient’s mind and body. Based on clinical phenotypic features, albinism can be divided into three broad categories. Ocular albinism (OA 1–2), in which patients experience only a decrease or lack of ocular pigmentation and exhibit varying degrees of visual impairment, photophobia, and other ocular symptoms; and oculocutaneous albinism (OCA 1–8), in which, in addition to the symptoms of deficient ocular pigmentation, low visual acuity, and photophobia, patients’ skin and hair are characterized by a significant lack of pigmentation. The first two types are known as nonsyndromic albinism. The other type is an albinism-associated syndrome, also known as syndromic albinism. This type of albinism is associated with defects in melanin and other cellular proteins. In addition to some degree of oculocutaneous albinism, patients suffer from immunocompromised Chediak Higashi syndrome type 1 (CHS-1) and Hermansky Pudlak syndrome types 1–10 (HPS 1–10), the latter of which leads to pulmonary fibrosis, cardiomyopathy, and hemorrhage [4, 5]. All albinisms are inherited in an autosomal recessive manner, except for OA, which exhibits X-linked recessive inheritance. Therefore, it is important to identify the genetic cause when clinically diagnosing the type of albinism, which will guide physicians in ruling out the risk of having an affected child for their patients. Epidemiologic survey studies have shown that OCA-1 is the main type of albinism in China, accounting for about 64.3%; OCA-2, OCA-4, and HPS-1 accounted for 11.7%, 15.6%, and 2.2%, respectively; and those who carried unknown mutations accounted for 6.2%, suggesting that there may be some as yet undiscovered causative genes [6, 7].

Because the clinical phenotype of OCA is difficult to distinguish and the causative genes are numerous and complex, gene diagnosis has become a useful tool for disease classification and genetic counseling. Traditional molecular testing methods such as first-generation sequencing are unable to comprehensively analyze all relevant genes or ensure timely etiological diagnosis. Whole-exome sequencing based on second-generation sequencing technology covers the exon region that mainly controls protein synthesis, which is associated with 85% of human disease-causing gene mutations. It is therefore widely used to screen for gene-related genetic diseases [8]. With the penetration and popularization of modern information technology, people can obtain, process, and utilize various information in medicine and medical management through computers, such as the prediction of pathogenicity of gene mutants, the prediction of drug stability, and the analysis of molecular dynamics, etc. [9, 10]. In this study, a family with albinism was analyzed using whole-exome sequencing to search for possible causative mutation loci, and computer tools were used to analyze the mutation loci to provide reliable eugenic genetic guidance for preventing the risk of albinism recurrence in this family.

## Object and methods

### Research object

The proband, is female, 31 years old, 11 weeks gestation, 1 pregnancy and 0 deliveries, non-consanguineous marriage. She was seen for poor vision bilaterally. Results showed 0.2 vision in both eyes, bluish sclera, slight nystagmus, yellowish hair, and fair skin all over the body, which was suspected to be albinism. General physical examination showed that the patient had mild early pregnancy reaction but normal fetal development, no history of vaginal bleeding, no history of viral infection or adverse drug use, no history of hypertension, heart disease, tuberculosis, diabetes mellitus, hepatic or renal disease, no history of surgery, and was generally in good condition. Her husband was in good health and had no genetic disorders. Prenatal serologic testing of the proband showed a threshold risk (1/953), fetal ultrasound showed no abnormalities, and noninvasive DNA testing showed no abnormalities. We examined the couple using the WES technique, and if mutations were found, they were family-verified using Sanger sequencing. The study was approved by the Ethics Committee of Medical Genetics and Prenatal Testing of Luoyang Maternal and Child Health Hospital, and both spouses signed an informed consent form.

### Methods

#### Extraction of peripheral blood DNA

After disinfecting the venous blood collection site of the family members, 2 – 4 mL of peripheral blood was collected and placed in an EDTA-K_2_ anticoagulant tube. We strictly follow the product manual to extract genomic DNA using the Tianan Genomic DNA Extraction Kit (Tiangen Biotech, China). Dissolve the extracted DNA in an appropriate amount of TE solution. After extraction, the concentration and purity of DNA were measured using a NanoDrop measuring instrument, and the qualified DNA was stored at −20 °C for future use.

#### Whole exome sequencing

We randomly fragmented the genomic DNA of family members into 180 – 280 fragments using a Covaris crusher and performed end repair, phosphorylation, and polyA addition using the Agilent SureSelect Human All Exon V5/V6 kit. The two ends of the fragments were connected to connectors to prepare a DNA library. We hybridized the library with a specific index with up to 543,872 biotin-labeled probes in the liquid phase and then captured 334,378 exons of 20,965 genes with streptomycin magnetic beads. After linear amplification by PCR, the library was tested. Then, Qubit 2.0 was used for preliminary quantification, and Agilent 2100 was used to detect the Insert size of the library. After the Insert size met expectations, qPCR was used to accurately quantify the library’s effective concentration (3 nmol/L) to ensure library quality. After passing the library inspection and meeting the standards, we used the Illumina NovaSeq 6000 sequencing platform to perform high-throughput sequencing on the enriched target fragments.

#### Using Sanger sequencing for family verification

We compared the sequencing data obtained with the reference sequence of the human genome GRCh37/hg19. If a suspicious mutation is detected, we will use Sanger sequencing to verify the site of the genomic DNA of family members. We designed primers and carried out PCR amplification. After the amplification product was purified by agarose gel electrophoresis, it was sequenced by an ABI3730xl genetic analyzer, and the sequencing results were analyzed by Chromas software, to determine the suspected pathogenic variation found by medical exon sequencing. (The primer sequence is as follows: *OCA2*-chr15-28090200-F: TCCATGCTGTTCTGCAATCC, *OCA2*-chr15-28090200-R: GCCCCACTTGTGTTTTTCTC; *OCA2*-chr15-28228553-F: TGAAGGACCAGTCACCTAAC, *OCA2*-chr15-28228553-R: AATCTCTGGGTTGCATGTGG; *OCA2*-chr15-28230316-F: GTAGCATGTACTGCAGTCAC, *OCA2*-chr15-28230316-R: CTAATGAAAGGCTGCCTCTG).

#### In silico analysis

According to the genetic test results, DNAMA software was used for conservative analysis of the mutation site, and PolyPhen-2 (http://genetics.bwh.harvard.edu/pph2/), Mutation Taster (http://www.mutationtaster.org/) and American College of Medical Genetics and Genomics (ACMG) gene mutation interpretation guidelines were used to analyze the pathogenicity of the mutation site. If the mutation causes changes in amino acids, we use SWISS-MODEL (https://swissmodel.expasy.org/) to predict the three-dimensional structure of the protein.

## Experimental results

### Clinical manifestations and WES test results of the patient

**Fig. 1:**
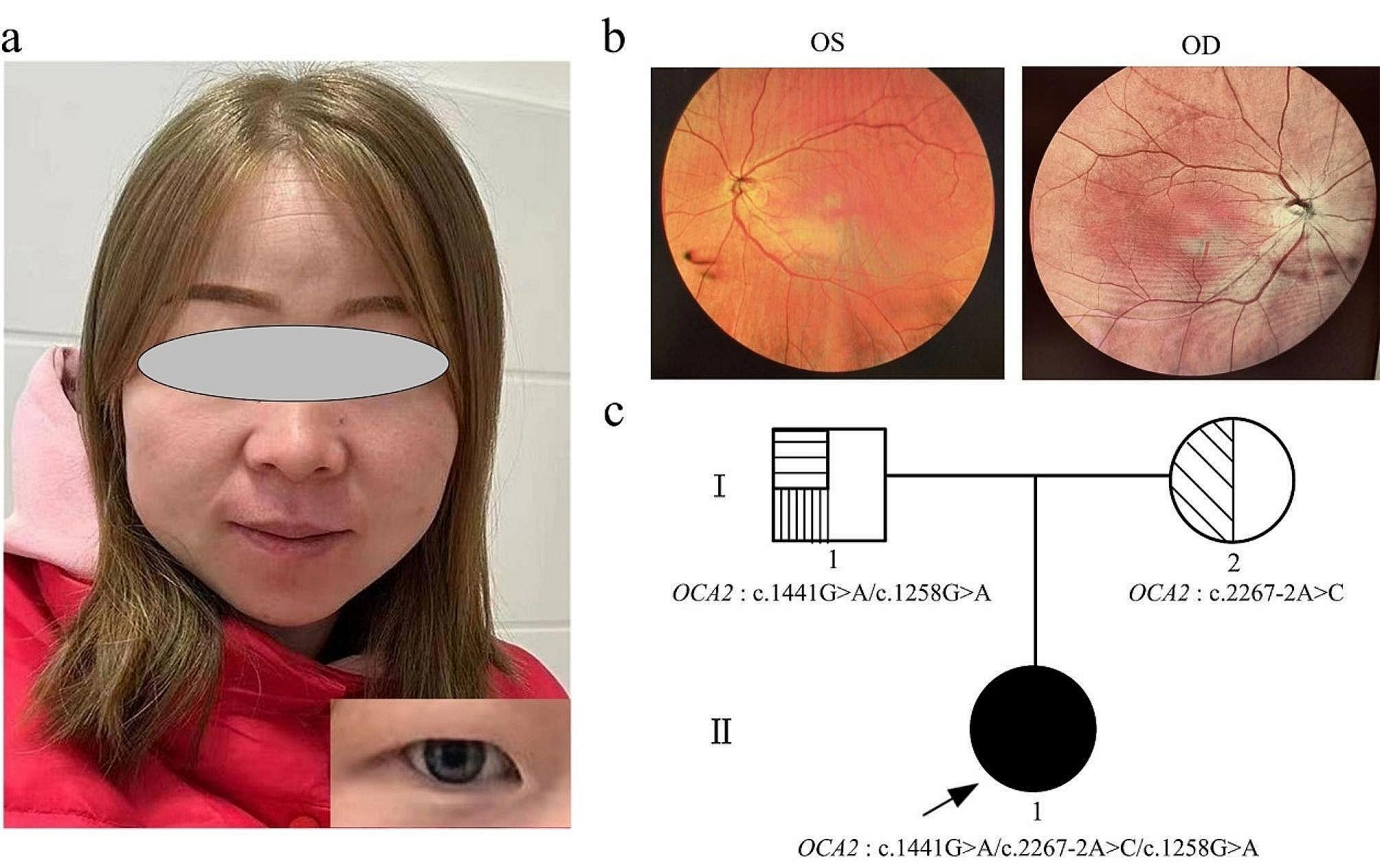
Basic clinical information about the patient. (**a**) Head and facial features of the patient. (**b**) Retinal examination results of the patient (OS: left eye; OD: right eye). (**c**) Family tree mapping of the patient

The clinical manifestations of the patient were white skin, yellow hair, a few freckles on the cheeks and bridge of the nose, decreased vision, and blue iris (Fig. 1a). Retinal examination showed that the fundus of both eyes of the patient was orange-red reflective, with little pigmentation, unclear optic disc borders, wet silken reflexes visible in the posterior pole of the retina, extensive atrophy of the retinal pigment epithelium in the periphery, and significant exposure of the networked choroidal vasculature (Fig. 1b).

WES testing results showed that the patient’s *OCA2* gene had three mutation sites: c.1441G > A (p.A481T) at chr15:28228553, c.2267-2 A > C at chr15:28090200, and c.1258G > A (p.G420R) at chr15:28230316 (Table 1). The patient was the proband in the family, and the family tree is mapped as shown in Fig. 1c.

**Table 1:** Results of gene mutation testing for probands

Gene	Transcript	The location of the genome	Zygotic type	Genetic mode	Diseases related to gene mutations
*OCA2*	NM_000275 exon14	chr15:28228553 c.1441G > A p.A481T	Heterozygote	AR	Skin or hair or eye pigmentation variant type I (MIM: 227,220); Oculocutaneous albinism type II (MIM:203,200)
*OCA2*	NM_001300984 exon22	chr15:28090200 c.2267-2 A > C	Heterozygote	AR	
*OCA2*	NM_000275 exon13	chr15:28230316 c.1258G > A p.G420R	Heterozygote	AR	

*Note* AD: Autosome dominant inheritance; AR: Autosome recessive inheritance

### Results of Sanger sequencing validation

**Fig. 2:**
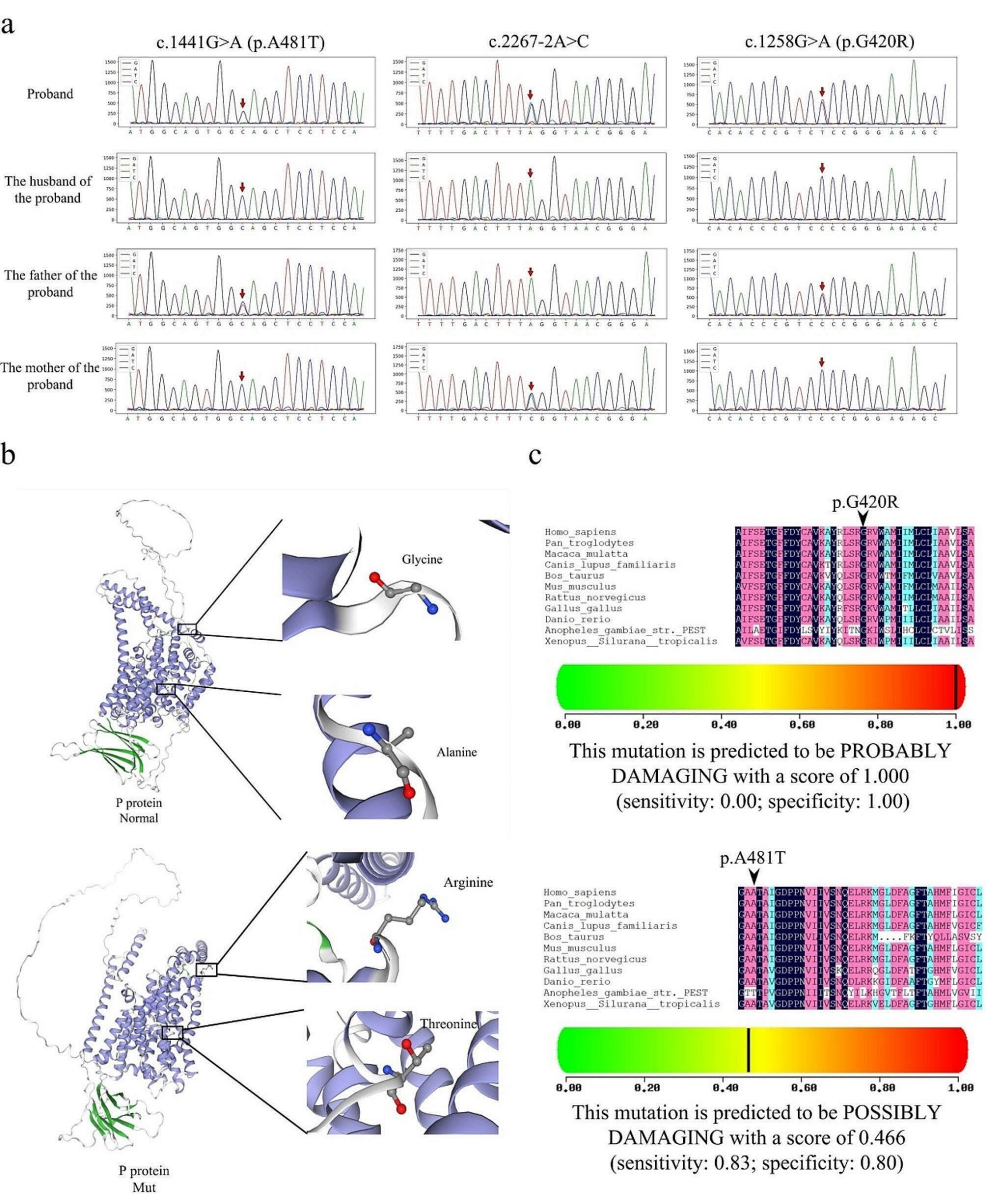
Validation of gene mutations and prediction of pathogenicity. (**a**) Sanger sequencing verification results of the proband’s couple and the proband’s parents. (**b**) 3D structure of P protein. The amino acid at position 481st changed from alanine to threonine, and the 420th amino acid changed from glycine to arginine (Seq identity: 99.76%, GMQE: 0.74). (**c**) Conserved analysis of gene mutation sites and PolyPhen-2 prediction analysis (The higher the score, the higher the pathogenicity)

Based on the results of the WES test, we used Sanger sequencing to validate the proband couple and the proband’s parents. The results showed that the c.1441G > A (p.A481T) and c.1258G > A (p.G420R) mutations in the proband were both paternal, whereas the c.2267-2A > C mutation was maternal; the husband of the proband was wild-type at three of the above loci (Fig. 2a; Table 2).

### Pathogenicity prediction of mutations

The mutation c.1258G > A (p.G420R) is located at the + 19 position of exon13, the base of c.1258 is mutated from G to A, and the amino acid glycine at amino acid 420 is mutated into arginine, which was a de novo mutation that had not been reported and was not included in the database. SWISS-MODEL was used to predict the 3D structure of proteins through the p.G420R mutation, we found that the residues of Pro420 participate in the coil of the 3D structure, and the prediction reliability is high. This is a highly conserved residue, and the ability of the human P protein to perform its function may be affected by mutations in this residue, which may ultimately lead to disease (Fig. 2b). In addition, through conservative analysis, this site was highly conserved in multiple species and the predicted result of PolyPhen-2 (with a score of 1.000) and Mutation Taster software was PROBABLY DAMAGING (Fig. 2c). However, according to the ACMG gene mutation interpretation guidelines, the mutation site met two pieces of evidence: PM2_supporting + PP3 (PM2_ Supporting: This mutation is currently not included in the normal population database; PP3: The predicted score for REVEL is 0.724, meeting the criteria for predicting harm), and was rated as of uncertain significance.

The mutation c.1441G > A (p.A481T) is located at the + 77 position of exon 14, and the c.1441 base is mutated from G to A. Amino acid alanine at position 481st is mutated to threonine. SWISS-MODEL predicted the three-dimensional structure of the protein after mutation, which the Pro481 residue remained in the alpha helix (Fig. 2b). Conservative analysis showed that this site was highly conserved across multiple species, and the result predicted by PolyPhen-2 software was POSSIBLY DAMAGING, with a score of 0.466, indicating that the change of this amino acid had certain pathogenicity (Fig. 2c). The mutation site was recorded in Clinvar and OMIM databases (rs74653330). The prediction of Mutation Taster showed that it was a “pathogenic mutation” (HGMD CM941112). The population frequency of this mutation site is 0.00799 (the maximum values of ESP6500, 1000 G, and EXAC_ALL), and the East Asian population frequency in the gnomAD database is 0.025. According to the ACMG reference guidelines, this site is a disease-related polymorphic site.

The mutation of c.2267-2 A > C is located at the − 2 position of Intron 21, c.2267-2 base mutated from A to C, this mutation did not undergo any amino acid changes and it has been included in the 1000 Genomes database (rs1470526281). Mutation Taster predicted that the results showed a “pathogenic mutation”. Referring to the ACMG gene mutation interpretation guide, this site meets two pieces of evidence: PVS_Moderate + PM2_Supporting (PVS_M: Classic splicing site mutation, predicted to not cause nonsense-mediated mRNA degradation, resulting in truncated protein length < 10%; PM2_Supporting: This mutation is currently not included in the normal population database) and is rated as Uncertain significance.

**Table 2:** Summary table of genetic validation of the parents of the proband

Gene	Mutational sites	Father of the proband	Mother of the proband
*OCA2*	c.1441G > A	Heterozygous mutation	Wild-type
*OCA2*	c.2267-2 A > C	Wild-type	Heterozygous mutation
*OCA2*	c.1258G > A	Heterozygous mutation	Wild-type

### Discussion

According to the occurrence of mutations, genes affecting ocular skin albinism (OCA) can be divided into: *TYR* (OCA1), *OCA2* (OCA2), *TYRP1* (OCA3), *SLC45A2* (OCA4), *SLC24A5* (OCA6), *C10ORF11* (OCA7), and *DCT* (OCA8), and one locus (OCA5) in consanguineous and sporadic albinism [11]. These genes encode enzymes or membrane transporters involved in melanin synthesis and tyrosine accumulation. Generally speaking, all types of albinism lack pigmentation, but their clinical manifestations vary depending on the type of disease.

OCA1 is common in Caucasian populations, and depending on whether there is a complete loss of tyrosinase activity can be categorized into two types, OCA1A and OCA1B, which are indistinguishable at birth. OCA1A with complete loss of tyrosinase activity is the most severe type. Patients also have a complete loss of skin and eye pigmentation and a decrease in visual acuity of up to 5%. OCA1B tyrosinase activity is significantly reduced, but not completely lost. The patient’s skin, hair, and eye pigmentation may increase with age and may be tanned [12]. OCA2 is highly prevalent in Africa and African Americans and is the most common type of albinism, accounting for about 50% of OCA worldwide [1, 13]. This type is mainly caused by mutations in the *OCA2* gene, which is located on chromosome 15q12-q13.1 and spans about 345 kb of genomic DNA in this region, including 30 exons. The *OCA2* gene encodes a holistic membrane protein (P protein) of the Na^+^/H^+^ countertransport protein family, which contains 12 transmembrane proteins α-the helical domain encodes 838 amino acids with a molecular weight of 110-KD. Available research evidence suggests that P proteins, as precursors of melanin synthesis, can bind to melanin and tyrosinase-associated proteins, participate in tyrosine transport, regulate the relative pH of melanosomes, and maintain skin and eye color [11, 14]. Typical OCA2 phenotypes include yellow hair, white skin, blue/beige/light brown iris, nystagmus, strabismus, and decreased visual sensitivity caused by abnormal optic nerve conduction pathways. Due to the lack of protection against melanin, skin pigmentation often accumulates into freckles or moles rather than being evenly distributed, making patients more susceptible to UV-induced skin cancer. OCA2-type patients are usually found to have a small amount of pigmentation in their hair and iris within 3 to 6 months of birth. As they age, the hair color of OCA2 patients increases and gradually manifests as yellow to brownish yellow, hence it is also known as incomplete albinism. Its eye symptoms may be slightly milder than OCA1A, but the skin, hair color, and clinical manifestations of the eyes are not easily distinguished from OCA1B [4, 15]. OCA3 was first found among black people. The pigment synthesized in the patient’s body is not black but brown. It is difficult to identify its clinical manifestations by appearance alone. It is characterized by light brown hair, light brown skin, blue/brown iris, nystagmus, and decreased vision. When a child with a very light skin color is born to parents with dark skin, its clinical manifestations will be relatively obvious [16]. OCA4 is more common in the Japanese population and presents with widespread depigmentation and ocular abnormalities, similar to OCA2 [17]. Patients with OCA6 have a great heterogeneity of skin phenotypes and a wide variation in hair color, from white to blonde up to dark brown, and affected patients have weak vision [18]. In addition to hypopigmentation, patients with OCA7 have significant ocular symptoms, including nystagmus, iris transparency, reduced visual sensitivity, and abnormal optic nerve cross-projections [19]. OCA8 is a mild albino phenotype that includes hypopigmentation of the hair and skin, moderate central fovea hypoplasia, nystagmus, and other manifestations [20]. In addition, the OCA5 locus has been localized to 4q24 in a closely related family in Pakistan, but the causative gene has not been identified [21]. Clinically, OCA types 5–8 are extremely rare in humans.

According to available data (UniProt, Clinvar, VarSome), a total of 642 mutations in the *OCA2* gene have been included, and these mutations are mainly missense mutations and loss-of-function mutations, which lead to structural changes in key regions of P protein, direct reduction in production, and decrease in quality and function, thus affecting downstream tyrosine transport, melanopsin regulation, and pigmentation. Unlike the *TYR* gene which has hotspot regions, the *OCA2* gene mutations in related diseases are more dispersed and no hotspot mutation regions have been identified [22].

In this study, we found three mutations in the patient: c.1441G > A (p.A481T), c.2267-2 A > c, and c.1258G > A (p.G420R), all of which were located in the *OCA2* gene. The p.A481T mutation is a polymorphic site, and homozygous mutations are not pathogenic. But when it forms a compound heterozygous mutations with another nonfunctional mutation, it has a mild phenotype, and carriers of heterozygous mutation have poor tolerance to sunlight. Threonine (ACC) replaces alanine (GCC) in the predictive lumen (or extracellular) ring of the P protein in the p.A481T mutation. Experiments transfected with p.A481T mutant *P* cDNA in mouse melanocytes showed that the A481T allele played about 70% of the role in melanin formation compared to wild-type human *P* cDNA, which is consistent with it being a relatively mild OCA2 mutant allele [23–25]. There were no relevant amino acid changes in the c.2267-2 A > C mutation, and we did not perform conserved analyses and protein 3D structure prediction. It has been reported in the literature that a change in the receptor site from AG to CG in intron 22 of OCA2 resulted in a predicted lack of splicing site recognition, which may have affected the splicing of OCA2 mRNA [26, 27]. In the p.G420R mutation, the Pro420 residue is located in the coil of the 3D structure of the protein, a site that is highly conserved in several species. Once mutated, it is likely to result in a conformational change in the protein. So far, no c.1258G > A (p.G420R) mutation has been reported, and it is considered to be a de novo mutation. Given the authority and professionalism of the ACMG guidelines, the current ratings for these three mutations, one polymorphic and two of uncertain clinical significance, do not meet the criteria for being potentially pathogenic/pathogenic and a clinical diagnosis cannot be based on this genetic test. The pathogenicity of the de novo mutation c.1258G > A (p.G420R) is difficult to clarify by computer prediction alone.

OCA2 albinism conforms to the classical autosomal recessive inheritance pattern and often occurs in people who are consanguineously married. If both spouses are heterozygous carriers, there is a 25% chance that the next generation will inherit both parents’ mutations and become patients, a 50% chance that they will become carriers, and a 25% chance that they will be completely normal [28, 29]. In the present study, the proband inherited heterozygous mutations from each of her parents, constituting compound heterozygous mutations that were responsible for her disease. c.1441G > A and c.1258G > A are inherited from the father, and c.2267-2 A > C is inherited from the mother. It is inferred that the two mutations inherited from the father are located on the same allele, either inherited at the same time or not. Since the proband carries three mutations at the same time, it suggests that the two mutations carried by her father himself are located in the same allele, which behaves in a cis arrangement, and therefore, her father does not develop the disease. Correspondingly, our follow-up results proved that the father of the proband was healthy. According to Mendelian laws of separation and free combination, the probability of the proband’s siblings carrying compound heterozygosity is 1/2 * 1/2, which is 25%. Unfortunately, the proband was the only child of her parents and had no siblings, and it is unclear whether siblings carrying the same mutation necessarily caused the disease. However, according to the law of autosomal recessive genetic disease, the proband marries a husband with a normal genotype, and the children born will be carriers of the disease genes with normal phenotype. Therefore, we do not need to screen for the *OCA2* gene in fetuses conceived by the proband. This not only saves the patient the cost of testing but also the expectation of delivering a healthy baby.

The limitations of this study are that we did not collect clinical information from more generations and without clinical data from siblings of proband, it is unclear whether siblings carrying the same mutation necessarily cause the disease. Therefore, it is necessary to collect more information about the population carrying the mutation and to perform in vitro functional validation of the locus, such as Minigene technology and Western Blotting, to verify the effect of the mutation on protein expression and to further elucidate the pathogenicity of the mutation.

For OCA disease, we recommend assisted reproductive conception or prenatal testing during pregnancy. If family members are known to carry the disease-causing mutation, carrier testing for high-risk relatives and prenatal screening for high-risk pregnancies can be performed. In addition, next-generation sequencing technologies, such as WES, are developing rapidly and have been widely used for assisted diagnosis of genetic diseases. It can cover currently known disease-related genes, rapidly discover disease-causing genes, and provide timely and accurate genetic counseling for patients’ families. The de novo mutation (c.1258G > A) identified in this study will also enrich the spectrum of mutations in OCA2-related genes and provide a good basis for physicians to diagnose the disease.

## Conclusions

We analyzed a case of albinism caused by a mutation in the *OCA2* gene by WES technology and identified a de novo mutation site (c.1258G > A), which together with two other mutation sites constitutes a compound heterozygous mutations as the cause of the disease. Our new finding enriches the database of genetic mutations in OCA2 albinism and will also provide reference value for genetic counseling, accurate diagnosis, and clinicopathological evaluation of this disease.

### Electronic supplementary material

Below is the link to the electronic supplementary material.

Supplementary Material 1

## Declarations

### Availability of supporting data

The datasets generated and analyzed during the current study are available in the Genome Sequence Archive for Human repository, and the accession number to datasets is HRA005353. It can be accessed from the following link: https://bigd.big.ac.cn/gsa-human/browse/HRA005353.

## Abbreviations

OCA: Oculocutaneous albinism
*OCA2*: OCA2 melanosomal transmembrane protein
OA: Ocular albinism
PCR: Polymerase chain reaction
WES: Whole exome sequencing
NGS: Next-generation sequencing
