# Supplementary material for: Genetic analysis of albinism caused by compound heterozygous mutations of the OCA2 gene in a Chinese family
Source: Hereditas. 2024 Feb 6;161:8. doi: 10.1186/s41065-024-00312-4 (PMC10845747; doi:10.1186/s41065-024-00312-4)
Supplement: Supplementary file 1 — Supplementary Material 1 [file 41065_2024_312_MOESM1_ESM.docx]

Appendix

Guidelines for interpreting mutations in ACMG related genes (partial content)

| **Pathogenic Mutation Site Grading Criteria** | |
| --- | --- |
| **Very strong** | **PVS1** is a malignant mutation site (null variant) on a known pathogenic gene in LOF (loss of function) (nonsense mutation, frameshift mutation, classical splice site ± 1 and 2 mutations, start codon change, loss of one or more exons). |
| **Strong** | **PS1** has the same amino acid changes as the previously identified pathogenic variant.  For example, changes in G>C or G>T within the same codon can lead to Val → Leu  Attention: Compared to changes in amino acid/protein levels, it is more important to pay attention to mutations that affect splicing.  **PS2**: Novel mutations carried by patients without a family history (verified by parents).  Note: Parental verification alone is not enough. Attention should also be paid to errors in egg donation, surrogacy, and embryo transfer.  **PS3**: In vivo and in vitro functional experiments have identified mutations that can lead to impaired gene function.  Note: Functional experiments need to be validated as effective, with repeatability and stability.  **PS4**: The frequency of mutations occurring in the affected population is significantly higher than that in the control population.  Note 1: Relative risk value or OR value can be selected for evaluation. It is recommended that sites with OR greater than 5.0 and confidence interval not including 1.0 be included in this item.  Note 2: Extremely rare variants may not have statistical significance in case-control studies, and this variant is prioritized in multiple patients with the same phenotype and not observed in the control group, which can serve as moderate level evidence. |
| **Moderate** | **PM1**: Non benign mutation sites located at mutation hotspots and/or critical and established functional domains (such as enzyme activity sites).  **PM2**: Mutations (or extremely low frequency loci in recessive genetic diseases) not found in the normal control population in ESP databases, thousand-person databases, and EAC databases.  Attention: The quality of insertion/deletion population data obtained from high-throughput sequencing is poor.  **PM3**: For recessive genetic diseases, multiple heterozygous pathogenic mutation sites are detected.  Note: This situation must be verified by the patient's parents or descendants.  **PM4**: Changes in protein length caused by loss of insertion/deletion or termination codons within non- repeating regions.  **PM5**: Newly discovered different missense mutations on amino acid residues previously identified as pathogenic for missense mutations.  For example, Arg156His has been reported as a pathogenic mutation, and now you have discovered Arg156Cys.  Attention: Compared to changes in amino acid/protein levels, it is more important to pay attention to mutations that affect splicing.  **PM6**: New emerging variants without parental sample validation. |
| **Supporting** | **PP1**: Mutation and disease are co-segregating in the family line (the variant is detected in multiple patients in the family line).  Note: As more segregation data become available, they can be used as stronger evidence.  **PP2**: Novel missense mutations in genes with a low rate of benign missense mutation sites and a common mechanism of disease.  Caveat: High-throughput sequencing yields insertion/deletion of population data of poor quality.  **PP3**: Multilineage computational evidence supporting deleterious effects on genes or gene products (conservation prediction, evolutionary prediction, splice site effects, etc.).  Note: Because many bioinformatics algorithms use the same or very similar inputs for prediction, each algorithm should not be considered an independent standard. PP3 can only be used once in the evaluation of any mutation site.  **PP4**: For a monogenic genetic disorder, the phenotype or family history of the variant carrier is highly compatible.  **PP5**: There are reports from reliable sources that the variant is pathogenic, but the evidence is not yet sufficient to support an independent laboratory evaluation. |
| **Benign Mutation Site Grading Criteria** | |
| **Stand-alone** | **BA1**: Variants with allele frequencies >5% in the ESP database, Thousands database, and EAC database. |
| **Strong** | **BS1**: Population carrier (Allele frequency) is greater than disease incidence.  **BS2**: For a disease that is fully penetrance at an early stage, the variant is found in healthy adults (pure for recessive diseases, heterozygous for dominant diseases, or X-linked hemizygous).  **BS3**: Variations confirmed to have no effect on protein function and splicing in both in vitro and in vivo experiments.  **BS4**: Lack of co-segregation among family members.  Attention: This section needs to consider complex diseases and penetrance issues. |
| **Supporting** | **BP1**: A disease is known to be caused by a truncated variant of a gene found in the gene of a missense variant.  **BP2**: For a fully penetrance dominantly inherited gene/disease, compound heterozygous disease-causing mutation loci are detected; Or, for any pattern of inheritance, a disease-causing mutation site is detected on the same chromosome.  **BP3**: In-frame deletions/insertions on repeat regions of unknown defined function.  **BP4**: Multilineage computational evidence showing no effect on genes or gene products (Conservative predictions, evolutionary predictions, splice site effects, etc.).  Note: Because many bioinformatics algorithms use the same or very similar entries for prediction, each algorithm cannot be treated as an independent standard. BP4 can only be used once in the evaluation of any one mutation site.  **BP5**: The variant found has an alternative molecular basis in disease.  **BP6**: A report from a reliable source considers the variant to be benign, but the evidence is not yet sufficient to support a laboratory-independent assessment.  **BP7**: The variant is synonymous and predicted not to affect splicing, and the corresponding nucleotide is not highly conserved. |
| **Pathogenicity classification** | **prerequisite** |
| **Pathogenic** | (1) 1 very strong (PVS1) and  (a) ≥1 strong (PS1-PS4) or  (b) ≥2 moderate (PM1-PM6) or  (c) 1 moderate (PM1-PM6) and 1 supporting (PP1-PP5) or  (d) ≥2 supporting (PP1-PP5)  (2) ≥2 strong (PS1-PS4) or  (3) 1 strong (PS1) and  (a) ≥3 moderate (PM1-PM6) or  (b) 2 moderate (PM1-PM6) and ≥2 supporting (PP1-PP5) or  (c) 1 moderate (PM1-PM6) and ≥4 supporting (PP1-PP5) |
| **Likely pathogenic** | (1) 1 very strong (PVS1) and 1 moderate (PM1-PM6) or  (2) 1 strong (PS1-PS4) and 1-2 moderate (PM1-PM6) or  (3) 1 strong (PS1-PS4) and ≥2 supporting (PP1-PP5) or  (4) ≥3 moderate (PM1-PM6) or  (5) 2 moderate (PM1-PM6) and ≥2 supporting (PP1-PP5) or  (6) 1 moderate (PM1-PM6) and ≥4 supporting (PP1-PP5) |
| **Benign** | (1) 1 Stand-alone (BA1) or  (2) ≥2 strong (BS1-BS4) |
| **Likely benign** | (1) 1 strong (BS1-BS4) and 1 supporting (BP1-BP7) or  (2) ≥2 supporting (BP1-BP7) |
| **Uncertain significance** | (1) Failure to meet the above criteria or  (2) Conflicting criteria for benign and pathogenicity |
